# Assessment of Pregnant Women’s Knowledge, Attitudes, and Habits Regarding Oral Health: Development and Validation of a Measurement Instrument

**DOI:** 10.3390/ijerph23030352

**Published:** 2026-03-11

**Authors:** Helena Glibotić Kresina, Ivana Dabo, Sandro Kresina, Elizabeta Dadić Hero, Sara Kresina, Danko Bakarčić, Martina Mavrinac, Neda Smiljan Severinski

**Affiliations:** 1 Department of Public Health, Teaching Institute of Public Health of Primorje-Gorski Kotar County, Krešimirova 52a, 51000 Rijeka, Croatia; ivana.dabo@zzjzpgz.hr; 2 Department of Public Health, Faculty of Health Studies, University of Rijeka, Viktora Cara Emina 5, 51000 Rijeka, Croatia; elizabeta.dadic.hero@uniri.hr; 3 Department of School and Adolescence Medicine, Teaching Institute of Public Health of Primorje-Gorski Kotar County, Krešimirova 52a, 51000 Rijeka, Croatia; sandro.kresina@zzjzpgz.hr; 4 Department of Family Medicine, Faculty of Medicine Rijeka, University of Rijeka, Braće Branchetta 20, 51000 Rijeka, Croatia; 5 Department of Psychiatry, Clinical Hospital Centre Rijeka, Krešimirova 42, 51000 Rijeka, Croatia; 6 Department of Social Medicine and Epidemiology, Faculty of Medicine Rijeka, University of Rijeka, Braće Branchetta 20, 51000 Rijeka, Croatia; 7 Faculty of Medicine Rijeka, University of Rijeka, Braće Branchetta 20, 51000 Rijeka, Croatia; sara.kresina@uniri.hr; 8 Department of Dentistry, Faculty of Dental Medicine, University of Rijeka, Krešimirova 40/42, 51000 Rijeka, Croatia; danko.bakarcic@fdmri.uniri.hr; 9 Department of Pediatric Dentistry, Clinical Hospital Centre Rijeka, Krešimirova 42, 51000 Rijeka, Croatia; 10 Medical Faculty of Pula, University Juraj Dobrila, Zagrebačka 30, 52100 Pula, Croatia; 11 Faculty for Speech and Language Therapy, University of Rijeka, Radmile Matejčić 2, 51000 Rijeka, Croatia; 12 Department of Gynecology and Obstetrics, Faculty of Medicine Rijeka, University of Rijeka, Vjekoslava Dujića 7, 51000 Rijeka, Croatia; nedass@uniri.hr; 13 Department of Obstetrics and Gynecology, University Hospital Rijeka, Vjekoslava Dujića 7, 51000 Rijeka, Croatia

**Keywords:** attitude, behavior, Croatia, knowledge, oral health, pregnancy, validation study

## Abstract

**Highlights:**

**Public health relevance—How does this work relate to a public health issue?**

- Lack of validated psychometric instruments for reliably assessing knowledge, attitudes, and oral hygiene practices in pregnant women.
- Lack of education of pregnant women about oral health.

**Public health significance—Why is this work of significance to public health?**

- Validation of a comprehensive instrument for knowledge, attitudes, and oral hygiene practices in pregnant women based on Standards for the selection of health Measurement Instruments (COSMIN) guidelines.
- Validated questionnaire is a reliable public health tool for systematic data collection, supporting targeted educational interventions to prevent caries and promote oral health among pregnant women.

**Public health implications—What are the key implications or messages for practitioners, policy makers and/or researchers in public health?**

- The validated instrument enables systematic data collection and evaluation of the effectiveness of specifically designed health-educational interventions to improve oral health in pregnant women.
- The integration of the comprehensive instrument into routine gynecological and dental prenatal care can facilitate the systematic identification of at-risk populations and the design of targeted educational programs.

**Abstract:**

Oral health during pregnancy is a critical factor in preventing caries in both mothers and children. Croatia currently lacks validated psychometric instruments for reliably assessing knowledge, attitudes, and oral hygiene practices in the general population. This study aimed to develop and provide initial psychometric evidence for a comprehensive instrument for this purpose in Croatia, with potential relevance for future cross-cultural adaptation. Following Consensus-based Standards for the selection of health Measurement Instruments (COSMIN) guidelines, an initial item pool was generated through literature review and expert consultation. The study was conducted from May 2024 to February 2025 in primary healthcare settings across Primorje-Gorski Kotar County (PGC). The validation study included 319 pregnant women. Exploratory factor analysis (EFA) revealed a six-factor structure with acceptable to strong factor loadings (ranging from 0.423 to 0.984). The instrument showed acceptable to good internal consistency (Cronbach’s α = 0.61–0.87) and excellent test–retest reliability (Pearson r = 0.993). Results indicated that knowledge about oral health was independent of attitudes and practices during pregnancy. Regression analyses were exploratory and did not provide evidence of predictive validity at this stage, suggesting that additional contextual and psychosocial factors should be considered in future modelling. The present study provides initial psychometric evidence supporting the proposed structure and reliability of the instrument; however, further confirmatory and longitudinal validation studies are required before broader implementation and generalization can be fully justified.

## 1. Introduction

Oral health is an integral component of systemic well-being, exerting a profound influence on overall quality of life [1,2,3,4]. Dental caries, as one of the most prevalent chronic diseases globally, remains a serious public health challenge despite its well-known etiology and effective preventive measures. It is associated with a range of negative health outcomes and is considered an important risk factor in the development of numerous chronic non-communicable diseases (NCDs), including childhood obesity, type 2 diabetes mellitus, cardiovascular diseases, and certain malignant neoplasms [2,5,6,7,8,9,10,11]. The connection between oral pathologies and NCDs is underpinned by shared modifiable risk factors, such as high-sugar diets, tobacco use, socioeconomic determinants, and chronic inflammatory pathways that may induce systemic effects. Consequently, the prevention of caries is fundamental not only for reducing the burden of oral disease but also for enhancing the general health status of the population [6,7,8,9].

In this context, the risk of developing early childhood caries is significantly influenced by pregnant women’s lack of knowledge regarding caries prevention, proper oral hygiene, and the importance of early dental check-ups during pregnancy [10]. Prioritizing oral healthcare during pregnancy and fostering evidence-based knowledge, attitudes, and practices among pregnant women is imperative, as these factors directly correlate with maternal health, obstetric outcomes, and the long-term oral health of the offspring [1,2,3,4].

Despite the scientifically confirmed importance of oral health care during pregnancy, studies indicate an insufficient level of health education during this period, resulting in inadequate knowledge, attitudes, and habits regarding the prevention of oral diseases in pregnancy [3,4,10,11].

An analysis of the available literature reveals that studies conducted within the Croatian population predominantly use questionnaires either adapted from prior research or author-constructed instruments whose formal psychometric validation has not been confirmed. These instruments have not undergone the necessary stages of psychometric assessment, including the evaluation of construct validity, internal consistency, and measurement stability. Therefore, the ability to objectively and consistently measure the knowledge, attitudes, and habits of pregnant women included in the research is limited [12,13,14,15,16,17]. Only in the study by Gavić et al. [14].was the questionnaire partially validated, specifically in the section related to knowledge about children’s oral health, whereas the sections addressing attitudes, behaviors, and practices of pregnant women did not undergo systematic validation. This represents an important methodological gap, as it cannot be reliably claimed that the instrument accurately measures the constructs of interest [15].

Compared with previously published Croatian studies, the international literature includes several instruments developed and validated to assess pregnant women’s knowledge, attitudes, and oral health behaviors [11,18,19,20,21,22,23]. These questionnaires are based on explicit theoretical models of health behavior, and their development included conducting factor analyses, evaluation of metric properties, and test–retest reliability assessments, thereby facilitating greater methodological standardization and international comparability [11,18,19,20,21,22,23]. The Republic of Croatia currently lacks population-adapted, psychometrically confirmed, and methodologically standardized questionnaires that would enable reliable data collection and the development of evidence-based interventions.

In addition to the systemic deficiency in educating pregnant women about oral health, the absence of validated instruments represents a significant barrier to achieving high-quality data collection and generating scientific evidence in national health practice [12,15,16]. Validated instruments are essential for the comprehensive analysis of factors influencing oral health and are crucial for enabling the development of targeted preventive and educational interventions [11,18,19,20,21,22,23]. To our knowledge, the present study represents one of the first attempts to systematically evaluate the knowledge, attitudes, and habits of pregnant women regarding oral health in the Republic of Croatia, thereby supporting the need for the development and cultural adaptation of a suitable measurement instrument. Health-related behaviors are commonly explained using theoretical models that emphasize the role of attitudes, perceived social norms, and perceived behavioral control. Central to this discourse is Ajzen’s Theory of Planned Behavior (TPB), which posits that behavior is not a simplistic function of knowledge; rather, it emerges from a synergistic interaction between an individual’s attitude toward a behavior, the prevailing subjective norms, and their perceived ease or difficulty in performing said behavior (perceived behavioral control). This framework has been widely applied in oral health research and provides a useful conceptual basis for understanding the oral health behaviors of pregnant women [24,25]. In the present study, TPB served not only as a conceptual background but also as a guiding framework for item generation and construct operationalization during questionnaire development. In line with this theoretical perspective, the present study focuses on assessing knowledge, attitudes, and perceived behavioral influences related to oral health during pregnancy.

The primary aim is to develop and rigorously validate an original standardized questionnaire designed to facilitate the reliable and objective assessment of knowledge, attitudes, and habits related to oral health in pregnancy. A validated instrument will enable systematic data collection as well as the evaluation of the effectiveness of specifically designed health-educational interventions.

Furthermore, a validated instrument may serve as a fundamental basis for the design and implementation of public health policies aimed at enhancing oral health during pregnancy, thereby contributing to improved overall health outcomes for both mothers and children.

## 2. Materials and Methods

This study aimed to develop and conduct an initial psychometric evaluation of the KAOH Questionnaire following established methodological recommendations for instrument development.

**Figure 1:**
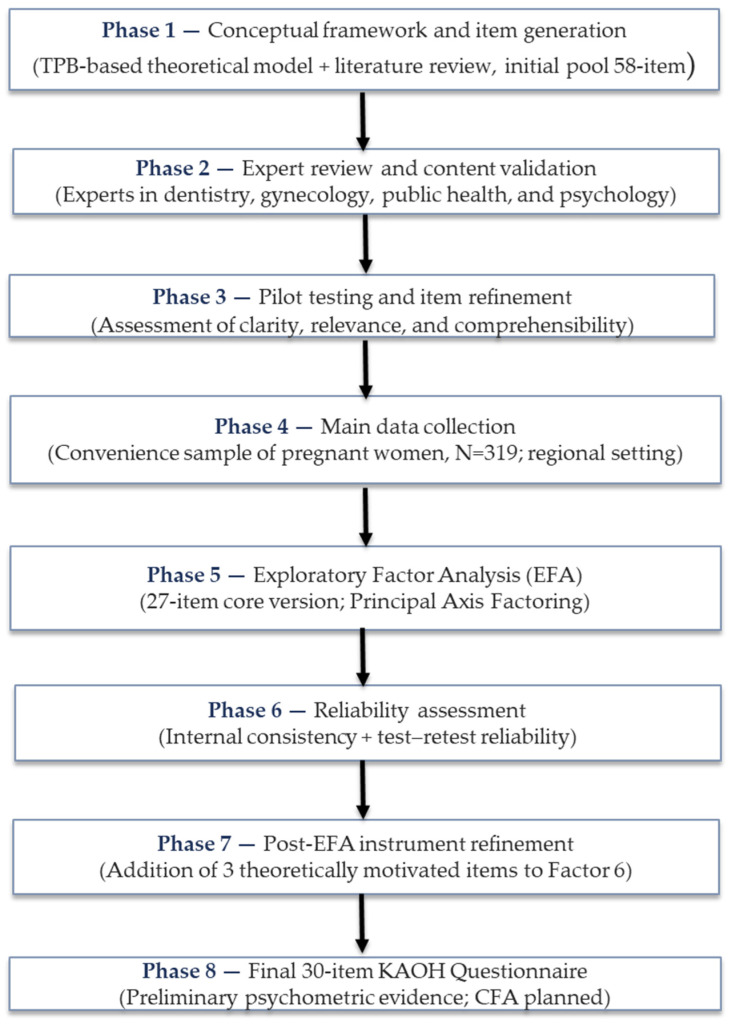
Phases of instrument development and staged psychometric validation of the KAOH Questionnaire.

The overall phases of instrument development and validation are summarized in Figure 1.

### 2.1. Participants

The validation of the questionnaire was conducted on a convenience sample of 319 pregnant women recruited between May 2024 and February 2025 in primary health care. The sample size was determined based on an assessment conducted by the National Public Health System. According to available data, 2332 children were born at the University Hospital Rijeka in 2020, while 2346 births were recorded in 2021. The average annual number of births for the observed two-year period was 2339. Based on estimated population size, and utilizing a standard sample size using G Power 3.1.9.7 software with a predefined confidence level and an acceptable margin of error, the necessary sample size for the questionnaire validation was calculated. The optimal number of female respondents, as determined by this calculation, was 300.

Participants completed the questionnaire anonymously during routine check-ups in gynecology primary healthcare practices across the PGC, irrespective of their gestational age. All questionnaires were coded, with access to the codes strictly limited to the research team. The Ethics Committee of the Health Center of PGC approved the study. Inclusion criteria mandated that all pregnant women be aged 18 years and older and provide their informed consent during their routine gynecological appointments within the selected practices. Pregnant women who did not provide informed consent or failed to complete the questionnaire fully were excluded from the study.

A formal invitation to participate in the study was extended via telephone to all primary gynecological practices within the Primorje-Gorski Kotar County (PGC). The final sample consisted of practices that voluntarily responded. Because participation in practices was voluntary, the final sample represents institutions that agreed to participate, which may introduce selection bias related to organizational characteristics, institutional motivation, or differences in patient engagement. Consequently, certain subgroups of pregnant women (e.g., those less engaged in prenatal care or with lower health literacy) may be underrepresented in the study sample. Exact participation rates at the individual participant level could not be calculated, as recruitment depended on attendance during routine clinical visits and voluntary consent at the time of data collection. The research protocol was approved by the Health Center PGC on 29 January 2024. The number of participants recruited from each practice was allocated proportionally based on the total number of pregnant women registered in that practice. Gynecologists and practice nurses were comprehensively informed about the study procedures before commencement. The questionnaires were distributed to the participants by the nurses upon arrival for their appointment, and respondents completed them in the waiting room either before or after their clinical check-up. To mitigate social desirability bias and ensure anonymity, completed instruments were deposited into a designated, sealed collection box, which was subsequently retrieved by the research team at weekly intervals.

Ethical rigor was strictly maintained throughout the investigative process. All participants provided written informed consent before enrollment. The study was conducted in full accordance with international bioethical standards, including the Declaration of Helsinki, ensuring the absolute confidentiality, privacy, and protection of all medical and personal data. The full version of the KAOH Questionnaire, Informed consent and Notice participation is available in the Supplementary Materials

### 2.2. Measurement Instrument

#### 2.2.1. Instrument Construction

The development of the Questionnaire on knowledge, attitudes, and oral hygiene habits of pregnant women (KAOH Questionnaire) is based on an analysis of scientific literature and existing validated instruments used in similar research [23,26]. The questionnaire was designed in collaboration with an interdisciplinary team: a pediatric dentist specialist (DB), an obstetrics and gynecology specialist (NSS), a public health specialist (HGK), and a graduate psychologist (MM). Based on a comprehensive review of evidence-based practices, the questionnaire was structured into three key domains: oral health knowledge, maternal and infant health attitudes, and prenatal hygiene behaviors. The development of the KAOH Questionnaire was explicitly guided by Ajzen’s Theory of Planned Behavior (TPB). During item generation, theoretical constructs were operationalized before statistical analysis to ensure conceptual alignment between the instrument and the underlying behavioral framework.

Items reflecting evaluative beliefs and emotional responses toward oral health behaviors were designed to capture the attitudinal component of TPB. Items addressing perceived expectations and influences from partners, family members, peers, and social surroundings were developed to operationalize subjective norms. Finally, items describing physical or situational barriers to oral hygiene during pregnancy were constructed to reflect perceived behavioral control.

Knowledge items were intentionally conceptualized as background determinants rather than direct behavioral predictors, consistent with TPB assumptions that knowledge influences behavior indirectly through attitudes, norms, and perceived control.

This theory-driven approach guided initial item development and informed the interpretation of the exploratory factor structure obtained in the subsequent analyses [22].

#### 2.2.2. Structure of the Questionnaire

The questionnaire consists of three parts:

Part One (16 items): This section encompasses sociodemographic data, prior oral health education, sources of information, dental service utilization, and reasons for selecting a dental clinic.

Part Two (12 items): This segment addresses pregnant women’s knowledge of proper nutrition, oral hygiene during pregnancy, infant oral care, and recommendations for utilizing dental services. For each item, the correct answer is predefined. The level of knowledge is quantified by the percentage of correct answers (ranging from 0% to 100%) and the total score (ranging from 0 to 23 depending on the complexity and number of correct options per item), categorized as follows: excellent (90–100%), score 21–23; very good (75–89.9%), score 18–20; good (60–74.9%), score 14–17; sufficient (50–59.9%), score 12–13; and insufficient (0–49.9%), score less than 12 points. The total score was calculated using weighted scoring, where certain items carried higher point values based on their complexity.

Part Three (30 statements): This section comprises the instrument’s validated core, focusing on pregnant women’s attitudes and oral hygiene habits. The questionnaire development process began with an initial pool of 58 items. Exploratory factor analysis (EFA) was conducted to refine the instrument, resulting in a reduced set of 27 items. Subsequently, three additional items were developed and incorporated to enhance the reliability and conceptual robustness of the identified factors. Consequently, the final version of the questionnaire consisted of 30 items. Importantly, the EFA was performed on the 27-item version, whereas the three additional items were introduced post hoc based on the factor-analytic findings to ensure more comprehensive construct coverage, particularly to strengthen the physiological barriers domain. The three additional items were introduced as part of an iterative refinement process aimed at improving conceptual coverage and internal consistency of Factor 6, which demonstrated comparatively lower reliability in the exploratory solution. Their inclusion was guided by theoretical considerations, expert consensus, and clinical relevance rather than data-driven optimization. Because these items were added after completion of the exploratory factor analysis, the factorial validity of the final 30-item version cannot be considered fully established at this stage and should be regarded as provisional pending confirmatory testing. Future studies should subject the expanded 30-item version to confirmatory factor analysis (CFA) to further evaluate its factorial structure and psychometric properties.

The validation process was conducted in two stages. First, we assessed face validity and comprehensibility through a pilot study with 15 pregnant women (excluded from the final analysis) to ensure the questions were clear and culturally appropriate. Subsequently, a multidisciplinary panel of experts in dentistry, gynecology, public health, and psychology evaluated the content validity, confirming that the items accurately reflected the core constructs of the Theory of Planned Behavior.

Validation procedures were concentrated on the third part of the questionnaire, which contains 27 statements (derived from an initial pool of 58) designed to assess pregnant women’s attitudes and habits regarding oral health. The statements are rated on a five-point Likert scale ranging from 1 (“strongly disagree”) to 5 (“strongly agree”). The items cover attitudes toward maintaining oral hygiene, the perceived importance of treating primary and permanent dentition, and the necessity of preventive dental examinations during pregnancy and early childhood. Demographic variables were collected using a separate author-designed form that may be adapted depending on future research aims.

### 2.3. Statistical Analyses

Validation of the KAOH Questionnaire on a sample size of 319 participants. The calculation was performed using the G*Power 3.1.9.7 software. The questionnaire was validated in accordance with the COSMIN guidelines, and the psychometric properties of the questionnaire were evaluated using descriptive statistics and exploratory EFA to assess structural validity. Reliability was examined through internal consistency and test–retest reliability, with internal consistency assessed using Cronbach’s alpha coefficient (α ≥ 0.70) and test–retest reliability evaluated using Pearson’s correlation coefficient over a two-week interval.

Statistical analyses of the obtained data included correlation analyses and regression modelling.

Because all variables deviated significantly from normal distribution (Kolmogorov–Smirnov tests, all *p* < 0.001), descriptive statistics are reported as median and interquartile range (IQR). Spearman’s rank correlations were used to examine associations between factors (F1–F6), knowledge, and age. A multiple linear regression was conducted to explore whether factors F1–F6 predicted the Knowledge score. All statistical analyses were conducted using JASP 0.19.1.0 (University of Amsterdam, Amsterdam, The Netherlands). Statistical significance was set at *p* < 0.05.

## 3. Results Validation of the KAOH Questionnaire According to the COSMIN Checklist

### 3.1. Structural Validity

An Exploratory Factor Analysis (EFA) was performed to assess the latent structure of the 27 items. Sampling adequacy was confirmed by a Kaiser–Meyer–Olkin value of 0.790 and a significant Bartlett’s Test of Sphericity (χ^2^ = 3718.640, df = 378, *p* < 0.001), indicating that the correlation matrix was suitable for factor analysis [27].

Principal axis factoring was selected as it is less dependent on normality assumptions than maximum likelihood methods and is commonly used when indicators are ordinal or non-normally distributed. The number of retained factors was determined using a combination of criteria (Kaiser criterion, scree plot inspection, and parallel analysis) to reduce reliance on any single heuristic and improve robustness of the factor-retention decision [28,29,30]. Given the theoretical and empirical expectation that latent constructs may be correlated, an oblique Promax rotation was applied [31,32].

A six-factor solution emerged, explaining 50.8% of the total variance. Eigenvalues for the retained factors were 5.153, 3.553, 2.652, 2.268, 1.862, and 1.373. The pattern matrix demonstrated a clearly interpretable factor structure with minimal cross-loadings. Factor loadings are reported as exploratory indicators of structural validity and are interpreted cautiously in accordance with the exploratory nature of the analysis. Uniqueness values ranged from 0.005 to 0.780.

Moderate correlations among the six factors (range: −0.488 to 0.420) further justified the use of oblique rotation. It should be emphasized that the reported factor structure refers exclusively to the 27-item exploratory version of the instrument. The additional three items included in the final questionnaire were not subjected to factor analysis within the present study.

#### Exploratory Factor Analysis

The six-factor solution was conceptually coherent:

F1: Perceived Importance of Primary Teeth and Early Dental Hygiene

F2: Oral Health Support in Pregnancy

F3: Negative Prenatal Dental Beliefs

F4: Perceptions of other women’s behavior

F5: Knowledge about caries consequences

F6: Fatigue, Nausea, and Oral Hygiene Discomfort

The pattern matrix supported the interpretability of the six factors, and most items displayed minimal cross-loadings. Uniqueness values ranged between 0.005 and 0.780, indicating acceptable levels of shared variance. The structure matrix confirmed that the observed relationships were consistent with theoretically distinct but related latent variables.

The results of the exploratory factor analysis, including factor loadings and internal consistency coefficients (Cronbach’s alpha), are presented in Table 1.

### 3.2. Reliability and Validity Analysis

Internal consistency of each factor was assessed using Cronbach’s alpha. The alpha values ranged from 0.61 to 0.87 across the six factors, indicating acceptable to very good internal consistency according to COSMIN criteria (α ≥ 0.70 is acceptable).

Factor loadings ranged from 0.452 to 0.893 in magnitude.

To obtain the total score of F1 (F1: Perceived Importance of Primary Teeth and Early Dental Hygiene), two items were recoded: items 1 (“Primary teeth do not need to be brushed because they will fall out and be replaced by permanent teeth”) and 3 (“Early childhood caries does not need to be treated until the tooth begins to hurt”).

To improve the internal consistency of Factor 6 (F6: Fatigue, Nausea, and Oral Hygiene Discomfort), three new items were subsequently added:

1. “I avoid brushing my teeth because the taste and smell of toothpaste make me feel nauseous and disgusted”.
2. “The sensitivity of my gums during pregnancy prevents me from maintaining regular oral hygiene”.
3. “I often forget to brush my teeth even though I am pregnant”.

A revalidation of the construct with confirmatory factor analysis (CFA) is planned for future studies. Consequently, the psychometric properties of the expanded 30-item version should be interpreted as preliminary until the factorial structure is confirmed using CFA in an independent sample.

The final version of the Questionnaire on knowledge, attitudes, and oral hygiene habits of pregnant women contains 30 items.

### 3.3. Test–Retest Reliability

Test–retest reliability after two weeks was strong: Pearson’s r = 0.993 (*p* < 0.001). Although this coefficient indicates high temporal agreement, interpretation should be cautious because very high correlations may partly reflect methodological factors such as the relatively short retest interval or response recall.

### 3.4. Face and Content Validity

The development of the instrument relied on a theoretical framework, expert feedback, and a pilot study to ensure content and face validity. As a result, the items are well-grounded in theory and appropriate for the local context.

### 3.5. Exploratory Associations (Hypothesis-Generating)

Exploratory correlations and regression analyses were performed to generate hypotheses regarding relationships between questionnaire factors and knowledge; these analyses were not intended to establish criterion-related validity or predictive validity at this stage.

### 3.6. Responsiveness and Measurement Error

Responsiveness and measurement error were not assessed in the present cross-sectional study and will be evaluated in future longitudinal research. Obtained factors for the KAOH Questionnaire (30 items).

Obtained Factors:

Factor 1: Perceived Importance of Primary Teeth and Early Dental Hygiene (α = 0.84)—5 items

This factor reflects mothers’ understanding of the importance of caring for children’s primary teeth and initiating oral hygiene early. Items with high positive loadings capture misconceptions (e.g., *“Baby teeth do not need to be brushed…”*), whereas negatively loading items represent accurate beliefs (e.g., *“It is very important to brush a child’s teeth as soon as they erupt.”*). Together, these indicate that the factor measures a continuum from misconceptions to accurate beliefs about early childhood oral health.

Factor 2: Oral Health Support in Pregnancy (α = 0.87)—4 items

This factor elucidates the construct of perceived social support regarding oral health education throughout the gestation period. High loadings for “spouse,” “parents,” “other pregnant women,” and “friends” indicate that participants believe multiple social agents should contribute to oral-health guidance. This suggests that pregnant women conceptualize oral health as a shared responsibility within their family and social network, not solely among healthcare professionals.

Factor 3: Negative Prenatal Dental Beliefs (α = 0.82)—5 items

The third factor refers to fears and misconceptions among pregnant women regarding the safety of dental treatments during pregnancy, which may lead to the avoidance of dental visits.

The items within the factor show moderate to high loadings (0.507–0.804), confirming the coherence of the construct. The highest loadings were recorded for statements indicating that women should avoid dental visits due to potential risks to the fetus (0.780) and that dental care should be postponed until after pregnancy (0.804). Similarly, the belief that visiting the dentist may harm the baby (0.797) strongly contributes to this factor. Items such as “A dentist should be visited only when a tooth hurts” (0.507) and “It is not advisable for a pregnant woman to maintain usual oral hygiene” (0.564) further highlight misconceptions related to dental care and preventive practices.

The high internal consistency (Cronbach’s α = 0.816) confirms that the items reliably measure negative prenatal dental beliefs.

Factor 4: Perceptions of other women’s behavior (α = 0.78)—7 items

This factor represents the perceived awareness and engagement of pregnant women in maintaining and promoting oral health, including their level of information and preventive practices during pregnancy.

All constituent items exhibited robust positive factor loadings, ranging from 0.452 to 0.700, indicating strong internal coherence. Higher scores reflect greater perceived awareness of the importance of maintaining good oral health. Items such as “Most pregnant women take care of their oral health” (0.628), “Most women visit their dentist regularly during pregnancy” (0.642), and “Other pregnant women are sufficiently informed about maintaining oral health” (0.700) indicate the importance of perceived knowledge and proactive behavior. Items with lower loadings (e.g., taking dietary supplements, 0.452; more frequent dental check-ups due to increased tooth sensitivity, 0.496) reflect practical aspects of pregnant women’s behaviors that are relevant to oral health, but they correlate less strongly with the overall perception of awareness and engagement in maintaining oral health. The internal consistency of this factor was satisfactory (Cronbach’s α = 0.78), indicating reliable measurement of the construct.

Factor 5: Knowledge about caries consequences (α = 0.71)—4 items

Factor 5 represents the perception of pregnant women regarding the impact of dental caries on overall health, emphasizing the importance of maintaining oral hygiene and timely management of dental problems during pregnancy.

All constituent items exhibited robust positive factor loadings, ranging from 0.497 to 0.758, indicating strong internal coherence. Higher scores reflect a greater perception of the consequences that caries can cause. Items such as “A decayed tooth of a child may cause decay in other teeth” (0.758) and “A tooth with caries can cause deterioration of neighbouring teeth” (0.681) highlight the perceived risk of local spread of dental disease. The item “Timely treatment of caries can prevent complications such as abscesses” (0.497) emphasizes the importance of preventive care and early intervention.

Factor 6: Fatigue, Nausea, and Oral Hygiene Discomfort Factor (α = 0.61)—5 items

This factor operationalizes the impact of gestational physiological symptoms—specifically fatigue and nausea—on the consistent maintenance of oral hygiene regimens. It measures the extent to which physical symptoms of pregnancy disrupt daily dental routines and prevent proper oral care.

All items showed positive loadings (0.499–0.984), with higher scores indicating greater perceived difficulty in maintaining oral hygiene due to pregnancy-related symptoms. The item “In the evening, I do not feel like brushing my teeth due to tiredness and sleepiness” (0.984) highlights the impact of fatigue, while the item “Nausea and vomiting during pregnancy prevent me from maintaining proper oral hygiene” (0.499) illustrates the effect of nausea as a barrier to oral care.

The internal consistency of the factor was moderate (Cronbach α = 0.61), indicating satisfactory, though somewhat lower, reliability in measuring the construct. To enhance the dimension’s internal consistency, the questionnaire was refined by incorporating three specific indicators: gum sensitivity, taste aversion to toothpaste, and pregnancy-related fatigue, bringing the final instrument to 30 items. The psychometric robustness of these new entries is slated for further validation in subsequent studies.

### 3.7. Descriptive Statistics

A total of 319 participants were included. Variables were non-normally distributed; therefore, medians and IQRs are reported. Knowledge had a median of 15.00 (IQR = 13–17). Age ranged from 21 to 45 years (Median = 33, IQR = 29–35). The distribution of scores for knowledge, attitudes, and habits, including median values, interquartile ranges, and the overall range, is summarized in Table 2.

### 3.8. Correlations

Correlations between factors, age, and knowledge were calculated using the Spearman correlation coefficient. Correlations between factors were low to moderate. Moderate correlations included:

F1–F3: r = −0.313, *p* < 0.001; F1–F5: r = 0.328, *p* < 0.001; F3–F5: r = −0.383, *p* < 0.001.

Results are presented in Table 3.

### 3.9. Regression Analyses

A multiple linear regression tested whether the following factors predicted the Knowledge score (*N* = 317): Factor 1: Perceived Importance of Primary Teeth and Early Dental Hygiene; Factor 6: Fatigue, Nausea, and Oral Hygiene Discomfort. The overall model was not significant: F (6, 310) = 1.02, *p* = 0.414. The predictors collectively accounted for 1.9% of the variance (R^2^ = 0.019, Adjusted R^2^ = 0.0003). None of the predictors significantly predicted knowledge (all *p* > 0.05). Partial and semipartial correlations were very small (r < 0.11).

The results indicate that knowledge operates largely independently from the attitudinal and behavioral factors captured by F1–F6 (Factor 1: Perceived Importance of Primary Teeth and Early Dental Hygiene; Factor 6: Fatigue, Nausea, and Oral Hygiene Discomfort). Given the non-significant model and minimal explained variance, the regression analysis should be interpreted as exploratory and hypothesis-generating rather than as evidence of predictive validity.

## 4. Discussion

The maintenance of oral health during the prenatal period is a critical determinant of both maternal well-being and optimal fetal development. Adherence to meticulous oral hygiene regimens and the early implementation of evidence-based preventive strategies are essential to mitigating the risk of adverse gestational complications. Furthermore, proactive dental care serves as a fundamental intervention for enhancing longitudinal health outcomes for the mother-child dyad [33]. Despite this established necessity, systematic public health research and standardized educational programs on oral health for pregnant women remain deficient in the Republic of Croatia, and there is no fully validated instrument to assess their knowledge, attitudes, and habits. Consequently, the primary objective of this research was to develop and psychometrically validate the first comprehensive measurement instrument—the Questionnaire on knowledge, attitudes, and oral hygiene habits of pregnant women (KAOH Questionnaire)—specifically adapted to the Croatian linguistic and cultural context. The construction and initial psychometric evaluation of this instrument represent a potentially important contribution to domestic scientific and public health practice.

### 4.1. Psychometric Properties of the Instrument

The psychometric validation of the newly developed instrument, KAOH Questionnaire, was conducted in strict adherence to the COSMIN taxonomy to ensure methodological rigor and transparency. EFA using Principal Axis Factoring revealed a six-factor structure explaining 50.8% of the total variance. Importantly, the present validation should be interpreted as an initial exploratory stage of instrument development, primarily aimed at establishing preliminary structural validity and reliability rather than providing a comprehensive psychometric evaluation. Sampling adequacy was supported by the KMO measure and Bartlett’s test. The EFA suggested a six-factor structure explaining 50.8% of variance, and internal consistency estimates ranged from acceptable to very good across most factors. These findings provide preliminary evidence of structural validity and conceptual differentiation among dimensions; however, broader construct validity (including convergent and discriminant validity) requires evaluation in future confirmatory studies. The range of factor loadings suggests clearly defined relationships between individual items and their latent dimensions, while the range of Cronbach’s alpha coefficients indicates acceptable to excellent internal consistency. The particularly high reliability observed for the first two factors confirms that pregnant women distinctly separate the importance of early oral hygiene from the role of social support during pregnancy. The interplay observed among factors F1, F3, and F5 (Factor 1: Perceived Importance of Primary Teeth and Early Dental Hygiene—Factor 3: Negative Prenatal Dental Beliefs—Factor 5: Knowledge about caries consequences) suggests a complex relationship between beliefs, emotional reactions, and health behaviors. A critical finding is that the knowledge dimension was not significantly associated with any other factor. This indicates that knowledge alone is an insufficient predictor of behavior; factors such as motivation, self-efficacy, and external barriers (e.g., fatigue and nausea) must also be considered. This aligns with the theoretical assertion that information provision, without a shift in attitude or perceived control, seldom leads to sustained behavioral modification [34]. Accordingly, the current findings should be interpreted as representing an early stage of psychometric validation rather than definitive confirmation of the instrument’s final measurement properties. It should be noted that the exploratory factor analysis was conducted on the 27-item core version, and therefore, the factorial validity of the final 30-item questionnaire remains provisional pending confirmatory factor analysis.

### 4.2. Critical Reflection on the Knowledge Dimension and Theoretical Implications

An important finding of this study is the lack of a statistically significant association between the knowledge dimension and any other analyzed factors. This underscores the premise that knowledge, in isolation, is an insufficient predictor of behavioral change. models. Such results are broadly consistent with established health behavior models, which posit that motivation, self-efficacy, and logistical barriers serve as critical mediators in habit formation [34]. While the regression model evaluating the relationships between these dimensions and the outcome variables did not reach statistical significance, this suggests that oral health behaviors in pregnant women are likely determined by a broader constellation of latent variables that current models may not fully encapsulate. The very small proportion of explained variance indicates that the proposed model did not adequately capture the determinants of knowledge within this sample. Therefore, the regression results should not be interpreted as evidence of predictive validity but rather as an indication that oral health knowledge and related behaviors are likely influenced by additional motivational, contextual, and psychosocial factors not included in the current exploratory framework.

These findings demonstrate a high degree of correspondence with the TPB. Within this instrument, TPB constructs were operationalized through:

Attitudes and Beliefs (F1 and F3),

Subjective Norms (F2 and F4),

Perceived Behavioral Control (F6—capturing barriers such as nausea and fatigue).

Importantly, these links were theoretically predefined during questionnaire construction rather than derived post hoc from statistical findings, thereby strengthening the instrument’s conceptual validity.

Given the exploratory nature of EFA, the mapping of factors onto TPB constructs should be interpreted as theoretically informed rather than confirmatory, and future CFA studies are required to further test this structure

The emergence of knowledge as an independent, unrelated dimension is consistent with the theoretical postulate that information provision alone, without changes in attitudes or perceived behavioral control, seldom leads to sustained behavioral modification.

### 4.3. Psychometric Stability and Instrument Refinement

The instrument demonstrated modest to robust reliability, characterized by high internal consistency and exceptional temporal stability. Cronbach’s alpha coefficients ranged from 0.71 to 0.87, indicating reliability ranging from acceptable to very good. The only exception was the sixth factor (Cronbach’s alpha = 0.61). In this case, the marginal value of Cronbach’s alpha coefficient reflects the inherent complexity and variability of physiological conditions in pregnant women, rather than indicating a weakness in the measurement instrument. To further enhance the reliability of this dimension (F6), three additional specific items were added (gum sensitivity, aversion to the taste of toothpaste, and forgetfulness due to pregnancy-related fatigue). The revised version of the questionnaire included 30 items.

The test–retest analysis confirmed exceptionally high stability of the instrument (Pearson r = 0.993). Although the test–retest coefficient was very high, this finding should be interpreted cautiously. The relatively short retest interval may have contributed to response stability through recall effects or response consistency unrelated to true construct stability. This consideration is particularly important given that one factor demonstrated only moderate internal consistency, suggesting that temporal agreement alone should not be interpreted as definitive evidence of measurement precision. Future studies should therefore consider longer retest intervals to better distinguish true construct stability from potential methodological influences. Content validity was affirmed through the multidisciplinary approach employed during item construction, involving experts from dentistry, gynecology, public health, and psychology. In line with psychometric best practices, exploratory factor analysis represents an initial stage of scale development aimed at identifying a plausible latent structure, whereas confirmatory factor analysis is required to test and verify the stability of the finalized model. Therefore, the current findings should be interpreted as providing preliminary psychometric evidence rather than definitive confirmation of the instrument’s final measurement properties. Although the observed factor structure suggests conceptual differentiation among constructs, convergent and discriminant validity were not formally assessed in the present study. Stronger evidence of construct validity will require confirmatory analyses and inclusion of external theoretically related measures in future research.

Despite the satisfactory psychometric structure of the instrument, the regression model utilized to assess the relationships between individual dimensions and the outcome variable did not achieve statistical significance. This outcome suggests that the analyzed models may not fully encompass the key determinants influencing pregnant women’s oral health behaviors. Although the results provide encouraging initial evidence, the instrument should not yet be considered fully validated, as confirmatory analyses and longitudinal evaluation remain necessary to establish structural stability and broader applicability.

Consequently, interpretations regarding relationships among constructs should remain cautious, as cross-sectional exploratory analyses cannot establish causal or predictive associations. Future studies employing longitudinal designs and theoretically expanded models will be required to adequately examine predictive relationships.

### 4.4. Comparison with Previous Research

One of the principal challenges in researching oral health among pregnant women internationally is the dearth of standardized and psychometrically validated instruments applicable across diverse linguistic and cultural settings. A review of the literature demonstrated no instrument currently exists in Croatia that has been reported as fully compliant with contemporary psychometric standards. In domestic literature, only the study by Gavić et al. describes the process of instrument construction and partial validation, yet construct validity was not confirmed, nor were COSMIN protocols applied. Other national studies have relied on instruments lacking confirmed psychometric properties [14,16,35]. In contrast, the international literature reports a greater number of studies employing validated instruments adapted for the pregnant population [11,18,19,20,23,35,36,37].

Some authors have developed entirely novel instruments, such as the CAPSOM questionnaire for the Mexican population (Ramírez-Trujillo), which demonstrated good content validity but lacked reporting of advanced psychometric indicators. Similarly, Gupta et al. developed an instrument for the Indian population, but without conducting a complete factor analysis and without test–retest assessment, which limits the available evidence regarding the reliability of the instrument [23,26].

Compared to the aforementioned studies, the KAOH Questionnaire provides structured early-stage psychometric evidence (structural validity and reliability) within the studied context; nevertheless, additional confirmatory testing is required before stronger comparative conclusions can be made. Adapting existing instruments is often beneficial for international comparisons, while the construction of new instruments allows for the more precise measurement of locally specific phenomena [38,39,40]. Therefore, the development and validation of this locally adapted instrument may represent an important scientific and public health contribution within the local context

### 4.5. Strengths of the Study

To our knowledge, few instruments in this field report validation procedures explicitly aligned with COSMIN principles across multiple domains, highlighting the need for transparent and staged validation approaches. A key strength of this study lies in its originality and its attempt to follow established methodological standards for early-stage psychometric validation. To our knowledge, this is among the first instruments in Croatia developed in alignment with COSMIN methodological principles for early-stage psychometric validation.

### 4.6. Implications for Clinical Practice and Public Health

By validating this questionnaire, a standardized questionnaire has been developed that may be used in future research and clinical settings. In gynecological and dental offices, it can serve as a quick screening tool to identify pregnant women with insufficient knowledge or risky habits who require additional education. At the public health level, the instrument can contribute to the development of new strategies for caries prevention and the promotion of oral health in pregnant women.

In the PGC, a lack of education on oral health within prenatal courses has so far been confirmed, so the results of this study can serve as an argument for introducing a mandatory oral health module in prenatal programs, in accordance with UNICEF recommendations [12,13].

However, these implications should be interpreted cautiously until further validation studies confirm the applicability of the instrument in broader and more diverse populations.

### 4.7. Limitations of the Study and Directions for Future Research

The study has certain limitations. While this study is subject to certain methodological considerations, primarily concerning the recruitment of participants from a single county, this focused approach facilitated a nuanced analysis of the demographic, socioeconomic, and health characteristics unique to the observed population. The use of a convenience sample recruited from a single geographic region substantially limits the external validity of the findings. Therefore, the results should be interpreted as preliminary psychometric evidence obtained within a specific regional context rather than as findings that can be directly generalized to the broader population of pregnant women in Croatia or other cultural settings. In addition, participation depended on the voluntary agreement of healthcare practices, which introduces the possibility of institutional-level selection bias. Practices that chose to participate may differ systematically from those that declined, potentially affecting the representativeness of the sample. Future studies should employ multicenter or probabilistic sampling strategies across geographically and culturally diverse populations to improve representativeness, strengthen external validity, and allow more robust cross-cultural testing of the instrument. Although such localized factors necessitate a cautious approach regarding international comparisons, our findings provide an initial model that warrants further testing and precise model that warrants further testing and validation across broader or culturally diverse contexts. An additional methodological limitation is that the exploratory factor analysis was conducted prior to the inclusion of three newly developed items. Although these items were theoretically motivated and intended to improve content validity, the factorial structure of the final 30-item instrument remains provisional until confirmed through confirmatory factor analysis. Future research will therefore include CFA on an independent sample as a continuation of the same validation process, enabling formal testing of model fit and structural stability. Due to the cross-sectional design, causal relationships between constructs cannot be inferred, and temporal dynamics of behavioral change remain outside the scope of the present study. Therefore, the present study should be viewed as an initial step within an ongoing validation process, and conclusions regarding widespread use or general applicability should remain cautious until further confirmatory evidence is obtained. Furthermore, measurement invariance across relevant demographic subgroups (e.g., age, parity, educational level, or other sociodemographic characteristics) was not examined in the present study due to sample size and the exploratory stage of development. Assessment of invariance represents an essential next step for establishing the stability and comparability of the instrument across different populations.

Data were collected via self-report, which carries the risk of socially desirable responses. Although anonymity procedures were implemented to reduce bias, self-report measures inherently carry the risk of socially desirable responding and recall bias, which may have influenced the observed responses. Although a test–retest analysis was performed, the basic design is cross-sectional, so it is not possible to fully assess the responsiveness of the instrument, i.e., its sensitivity to changes over time. In addition, the regression model demonstrated limited explanatory power, which restricts the interpretation of relational patterns among constructs. As a result, conclusions regarding predictive or explanatory relationships should be considered preliminary and hypothesis-generating only. Therefore, the current findings should be considered preliminary from a structural validity perspective until confirmatory analyses are performed on the final 30-item version. Additionally, the very high test–retest coefficient should be interpreted with caution, as the relatively short retest interval may have increased response consistency beyond true construct stability.

Future research will include CFA, while responsiveness and measurement error will be evaluated using longitudinal data. Furthermore, future research should include a longitudinal approach, tracking changes in knowledge, attitudes, and habits from the beginning of pregnancy through the postpartum period, as well as comparing the dental status of pregnant women with their knowledge, education, and habits. Additionally, the instrument could be used in randomized controlled trials to evaluate different educational interventions. Comprehensive validation will require additional studies incorporating confirmatory factor analysis, convergent and discriminant validity testing, criterion-related validation, and measurement invariance analyses in independent and more diverse samples. These methodological limitations should be carefully considered when interpreting the findings, and the results should therefore be viewed as preliminary and primarily exploratory rather than definitive.

## 5. Conclusions

The KAOH Questionnaire provides initial psychometric evidence supporting a six-factor structure and acceptable internal consistency in a regional sample of pregnant women in Croatia. While these findings are encouraging, the instrument should be considered at an early stage of validation. Further confirmatory factor analysis, broader construct validity assessment (including convergent and discriminant validity), measurement invariance testing, and longitudinal evaluation are required before the instrument can be considered fully validated or recommended for widespread implementation.

Comparable instruments have been developed internationally and contribute important insights into oral health research during pregnancy. However, differences in validation approaches and reporting standards across studies make direct methodological comparisons challenging. The present study contributes additional early-stage psychometric evidence within the Croatian context, while emphasizing the need for continued confirmatory validation.

A key finding of this study—the independence of the knowledge dimension from attitudinal factors—suggests that future public health strategies may need to move beyond information dissemination alone and additionally address barriers such as physical discomfort, social support, and motivation. Reliability findings, particularly test–retest stability, should be interpreted within the context of the study design, and future studies with longer retest intervals are needed to further confirm temporal stability.

The integration of this questionnaire into routine gynecological and dental prenatal care may facilitate the systematic identification of at-risk populations and support the development of targeted educational programs. Given its initial methodological foundation and encouraging reliability indicators, the instrument also shows potential for future international comparative research, provided that additional cross-cultural validation studies are conducted. Nevertheless, the current findings should be interpreted as preliminary, as broader application requires further confirmatory validation in independent and geographically diverse samples. Overall, the present study represents an initial step within a broader validation process. Ultimately, the KAOH Questionnaire may provide a foundation for future evidence-based policies aimed at achieving long-term improvements in oral and general health outcomes for both mothers and children in Croatia.

## Figures and Tables

**Table 1:** Exploratory Factor Analysis: Factor Loadings and Cronbach’s Alpha.

Factor/Item Description	Item No.	Factor Loading	Cronbach’s α
F1. Perceived Importance of Primary Teeth and Early Dental Hygiene			0.84
Primary teeth do not need to be brushed because they will fall out and be replaced by permanent teeth.	1	0.831	
It is very important to begin brushing a child’s teeth as soon as they erupt.	2	−0.820	
Early childhood caries does not need to be treated until the tooth begins to hurt.	3	0.841	
It is crucial to care for a child’s dental health starting from birth.	4	−0.764	
Tooth decay in children can lead to long-term diseases of other organs.	5	−0.423	
F2. Oral Health Support in Pregnancy			0.87
Question: Besides dentists and physicians, who else should educate pregnant women on oral hygiene and dental protection during pregnancy?			
Spouse.	6	0.876	
Parents.	7	0.893	
Other pregnant women.	8	0.662	
Friends and acquaintances.	9	0.709	
F3. Negative Prenatal Dental Beliefs			0.82
Regardless of whether a woman is pregnant or not, a dentist should be visited only when a tooth hurts.	10	0.507	
I avoid visiting the dentist during pregnancy to prevent possible infection of the fetus.	11	0.780	
Pregnant women should not visit the dentist because it may harm the baby.	12	0.797	
Dental care should be postponed until after pregnancy.	13	0.804	
It is not advisable for a pregnant woman to maintain her usual oral hygiene.	14	0.564	
F4. Perceptions of other women’s behavior			0.78
Most pregnant women take care of their oral health.	15	0.628	
Most women visit their dentist regularly during pregnancy.	16	0.642	
Other pregnant women are sufficiently informed about maintaining oral health.	17	0.700	
During pregnancy, women seek more information about oral health.	18	0.505	
During pregnancy, women pay more attention to their oral health.	19	0.690	
Pregnant women take supplements that support the child’s dental health.	20	0.452	
Because teeth are more sensitive in pregnancy, most women attend dental checkups more often.	21	0.496	
F5. Knowledge about caries consequences			0.71
Dental caries may cause an infection that can spread to other parts of the child’s body.	22	0.517	
A decayed tooth of child may cause decay in other teeth.	23	0.758	
A tooth with caries can cause deterioration of neighboring teeth.	24	0.681	
Timely treatment of caries can prevent complications such as abscesses.	25	0.497	
F6. Fatigue, Nausea, and Oral Hygiene Discomfort			0.61
In the evening, I do not feel like brushing my teeth due to tiredness and sleepiness.	26	0.984	
Nausea and vomiting during pregnancy prevent me from maintaining proper oral hygiene.	27	0.499	

Note: Factor analysis was conducted on the 27-item core; items 28–30 were added to the final instrument based on qualitative refinement.

**Table 2:** Descriptive Statistics for Study Variables (Median, Interquartile Range, and Range).

Variable	Range (Min–Max)	Median	Interquartile Range (IQR)	Interpretation
F1 Perceived Importance of Primary Teeth and Early Dental Hygiene	4.00–20.00	5.00	4.00–8.00	Low
F2 Oral Health Support in Pregnancy	4.00–20.00	11.00	8.00–14.00	Medium
F3 Negative Prenatal Dental Beliefs	0.00–25.00	6.00	5.00–10.00	Low
F4 Perceptions of other women’s behavior	7.00–35.00	23.00	21.00–25.00	Medium to High
F5 Knowledge about caries consequences	4.00–20.00	17.00	15.00–20.00	High
F6 Fatigue, Nausea, and Oral Hygiene Discomfort Factor	0.00–10.00	2.00	2.00–3.00	Low
Knowledge	5.00–22.00	15.00	13.00–17.00	Medium
Age (years)	21.00–45.00	33.00	29–35	–

**Table 3:** Correlations between factors, knowledge, and age presented with Spearman correlation coefficients and *p* values in brackets below.

Variable	F1—Perceived Importance of Primary Teeth and Early Dental Hygiene	F2—Oral Health Support in Pregnancy	F3—Negative Prenatal Dental Beliefs	F4—Perceptions of Other Women’s Behavior	F5—Knowledge About Caries Consequences	F6—Fatigue, Nausea, and Oral Hygiene Discomfort Factor	Knowledge
F2 Oral Health Support in Pregnancy	0.063 (0.264)	1					
F3 Negative Prenatal Dental Beliefs	−0.313 (<0.001)	0.080 (0.152)	1				
F4 Perceptions of other women’s behavior	0.079 (0.160)	0.131 (0.019)	0.014 (0.807)	1			
F5 Knowledge about caries consequences	0.328 (<0.001)	−0.007 (0.903)	−0.383 (<0.001)	0.053 (0.350)	1		
F6 Fatigue, Nausea, and Oral Hygiene Discomfort Factor	−0.152 (0.007)	−0.023 (0.689)	0.062 (0.272)	−0.073 (0.192)	−0.058 (0.299)	1	
Knowledge	0.009 (0.869)	−0.072 (0.200)	0.015 (0.784)	0.056 (0.321)	−0.014 (0.810)	−0.061 (0.279)	1
Age	−0.110 (0.066)	0.045 (0.455)	0.031 (0.603)	0.051 (0.396)	−0.026 (0.669)	0.035 (0.553)	−0.079 (0.184)

Correlations involving Knowledge and Age were weak and nonsignificant (*p* > 0.05).

## Data Availability

The datasets used and/or analyzed during the current study are available from the corresponding author on reasonable request.

## Supplementary Materials

The following supporting information can be downloaded at: https://www.mdpi.com/article/10.3390/ijerph23030352/s1, The following materials are supplemented: Questionnaire on knowledge, attitudes, and oral hygiene habits of pregnant women. Informed consent. Notice participation and informed consent.

## Abbreviations

The following abbreviations are used in this manuscript:

CFA	Confirmatory Factor Analysis
COSMIN	Consensus-based Standards for the selection of health Measurement Instruments
EFA	Exploratory Factor Analysis
IQR	Interquartile Range
KAOH	Questionnaire on knowledge, attitudes, and oral hygiene habits of pregnant women
NCDs	Non-communicable Diseases
PGC	Primorje-Gorski Kotar County
TPB	Theory of Planned Behavior
