# Supplementary material for: Assessment of Pregnant Women’s Knowledge, Attitudes, and Habits Regarding Oral Health: Development and Validation of a Measurement Instrument
Source: Int J Environ Res Public Health. 2026 Mar 11;23(3):352. doi: 10.3390/ijerph23030352 (PMC13026591; doi:10.3390/ijerph23030352)
Supplement: Supplementary file 1 [file ijerph-23-00352-s001.zip › ijerph-4164411-supplementary.pdf]

Questionnaire on knowledge, attitudes, and oral hygiene habits of pregnant women

Code:______________________

Teaching Institute of Public Health of Primorsko-Goranska County
Clinical Hospital Center Rijeka
Faculty of Dental Medicine, University of Rijeka
Faculty of Medicine, University of Rijeka

Dear Participant,

Thank you for agreeing to take part in this study, which aims to explore pregnant women's knowledge, attitudes, and habits related to oral health.
Please read the instructions carefully before answering the questions. You may respond by selecting the option that best reflects your answer or by providing a written response where required.
All your personal information will remain strictly confidential.

GENERAL QUESTION:

1. Have you previously attended a session or module on oral health as part of a pregnancy course?
a) Yes
b) No

2. During your visit to your chosen dentist, did you receive advice on maintaining your oral health?
a) Yes
b) No

3. During your visit to a doctor of another specialty, did you receive advice on maintaining your oral health?
a) Yes
b) No

4. If you answered YES to the previous question, please specify the medical professional's specialty from whom you received information on maintaining your oral health (You may select more than one option):
a) Family medicine doctor
b) Gynecologist
c) Pediatrician

d) Internist
e) Doctor of another specialization (________________)

5. During your visit to your chosen dentist, did you receive advice on maintaining your child's oral health?
a) Yes
b) No

6. During your visit to a doctor of another specialty, did you receive advice on maintaining your child's oral health?
a) Yes
b) No

7. If you answered YES to the previous question, please specify the medical professional's specialty from whom you received information on maintaining your child's oral health (You may select more than one option):
a) Family medicine doctor
b) Gynecologist
c) Pediatrician
d) Internist
e) Doctor of another specialization (________________)

8. If you did not receive information on maintaining oral health from a medical doctor, did you obtain information on maintaining your oral health from another source?
a) Yes
b) No

9. If the answer is YES, please state the source (You may select more than one option):
a) Family
b) Friends
c) Social networks (internet, Facebook, Instagram, TikTok, etc.)
d) Media – TV, radio, print
e) Someone else ______________

10. If you did not receive guidance on maintaining your child's oral health from a medical doctor, did you receive this information from another source?
a) Yes
b) No

11. If the answer is YES, please state the source (You may select more than one option):
a) Family
b) Friends or acquaintances
c) Social networks
d) Media – TV, radio, print
e) Someone else __________________

12. What are the main reasons for your dental visits (You may select more than one option)?

a) Toothache

b) Fear of my teeth deteriorating

c) I care about my overall health, so I visit the dentist twice a year, even when my teeth do not hurt

d) Something else ____________________________

13. How satisfied are you with the services provided by your dentist?

a) Very dissatisfied

b) Dissatisfied

c) Neither

d). Satisfied

e) Very satisfied

14. What are the most common reasons you visit your dentist during pregnancy? (You may select more than one option.)

a) Toothache or dental pain

b) Concern about my teeth deteriorating

c) Concern for my baby's health due to potential complications from dental issues

d) To feel reassured and maintain overall health

e) I avoid visiting the dentist during pregnancy

f) Other (please specify)

I KNOWLEDGE:

1. Which of the following foods can most increase the incidence of dental caries:

a) Sweets and foods that stick to the teeth

b) Fruit

c) Vegetables

d) Dairy products

e) Water

2. How long is it recommended to brush the teeth?

a) 1 minute

b) 2–3 minutes

c) 5 minutes

d) 10 minutes

e) It is not important how many minutes teeth are brushed

3. How often should a toothbrush be changed?

a) Every month

b) Every 3 months

c) Every 6 months

d) Every 8 months

e) Every 12 months

4. How often should a person with healthy teeth visit the dentist for a check-up?
a) Every 3 months
b) Every 6 months
c) Once a year
d) Once every two years
e) In case of tooth pain

5. Which nutrients that we take into the body through food are considered responsible for the development of caries:
a) Sugars
b) Proteins
c) Fats
d) Minerals
e) Water

6. When is it recommended to brush your teeth?
a) After every meal
b) In the morning and before bedtime
c) Only before bedtime
d) Whenever your teeth feel dirty
e) Only in the morning

7. How many primary (baby) teeth do we have?
a) 16
b) 20
c) 24
d) 30
e) 32

8. If dental treatment or management of other oral health issues is required during pregnancy, during which trimester is it considered safest for the fetus to undergo such treatment?
a) In the first 3 months of pregnancy
b) From the 4th to the 6th month of pregnancy
c) In the last third of pregnancy
d) At the end of pregnancy
e) All of the above

9. In the case of a dental emergency (toothache, abscess, or dental injury), a pregnant woman:
a) Should see a dentist, but only after the sixth month of pregnancy
b) Should see a dentist, but not before the fourth month of pregnancy
c) Should seek treatment immediately, regardless of the stage of pregnancy
d) Must wait until after childbirth
e) Should avoid visiting the dentist during pregnancy

10. When is it most important to brush your teeth during pregnancy?
a) After waking up and before going to sleep
b) Only after waking up
c) Only before going to sleep
d) It is not recommended to brush teeth during pregnancy
e) After every-meal

11. Immediately after experiencing morning sickness or vomiting:
a) You should eat a nutritious meal
b) You should brush your teeth thoroughly
c) You should rinse your mouth with water
d) All of the above
e) No action is necessary

12. Please select the answer you believe is correct.

	TRUE (T)	FALSE (F)
A pregnant woman's teeth are more susceptible to decay.	T	F
Breastfeeding does not affect the health of a child's teeth.	T	F
Children's caries do not need to be treated until the tooth starts to hurt.	T	F
During pregnancy, it is necessary to take even greater care of dental health and hygiene than before.	T	F
During pregnancy, calcium supplements are recommended to help strengthen bones and teeth.	T	F
Only sweet foods can cause caries.	T	F
Still water cannot cause caries.	T	F
Primary teeth do not need to be treated if they have caries, as they will fall out anyway.	T	F
Primary teeth do not hurt with the same intensity as permanent teeth.	T	F
Immediately after the child is born, the mother transfers bacteria from her mouth to the child's mouth.	T	F
If a pregnant woman has a toothache, she may take an antibiotic without consulting a doctor to calm the infection.	T	F

II ATTITUDES, HABITS:

For the following questions, answer by circling the number that best corresponds to your opinion and attitudes.

1. In your opinion, besides dentists and physicians, who else should educate pregnant women on oral hygiene and dental protection during pregnancy?

	Strongly disagree	Mostly disagree	Neutral	Mostly agree	Strongly agree
Spouse.	1	2	3	4	5
Parents.	1	2	3	4	5
Other pregnant women	1	2	3	4	5
Friends and acquaintances.	1	2	3	4	5

2. Please indicate your opinion on the following statements:

	Strongly disagree	Mostly disagree	Neutral	Mostly agree	Strongly agree
Primary teeth do not need to be brushed because they will fall out and be replaced by permanent teeth.	1	2	3	4	5
It is very important to begin brushing a child's teeth as soon as they erupt.	1	2	3	4	5
Early childhood caries does not need to be treated until the tooth begins to hurt.	1	2	3	4	5
Dental caries may cause an infection that can spread to other parts of the child's body.	1	2	3	4	5
A decayed tooth of child may cause decay in other teeth.	1	2	3	4	5
It is crucial to care for a child's dental health starting from birth.	1	2	3	4	5

3. Please provide your opinion on the following statements and select a number from 1 to 5 that best reflects your view."

	Strongly disagree	Mostly disagree	Neutral	Mostly agree	Strongly agree
A tooth with caries can cause deterioration of neighboring teeth.	1	2	3	4	5
Timely treatment of caries can prevent complications such as abscesses.	1	2	3	4	5
In my opinion, tooth decay in children can lead to long-term diseases of other organs.	1	2	3	4	5

Regardless of whether a woman is pregnant or not, I believe that a dentist should be visited only when a tooth hurts.	1	2	3	4	5
I avoid visiting the dentist during pregnancy to prevent possible infection of the fetus.	1	2	3	4	5
Pregnant women should not visit the dentist because it may harm the baby.	1	2	3	4	5
Dental care should be postponed until after pregnancy.	1	2	3	4	5
It is not advisable for a pregnant woman to maintain her usual oral hygiene.	1	2	3	4	5
In the evening, I do not feel like brushing my teeth due to tiredness and sleepiness.	1	2	3	4	5
Nausea and vomiting during pregnancy prevent me from maintaining proper oral hygiene.	1	2	3	4	5
I avoid brushing my teeth because the taste and smell of toothpaste make me feel nauseous and disgusted.	1	2	3	4	5
The sensitivity of my gums during pregnancy prevents me from maintaining regular oral hygiene.	1	2	3	4	5
I often forget to brush my teeth, regardless of being pregnant.	1	2	3	4	5

4. Please provide your opinion on the behavior of other pregnant women and parents:

	Strongly disagree	Mostly disagree	Neutral	Mostly agree	Strongly agree
Most pregnant women take care of their oral health.	1	2	3	4	5
Most women visit their dentist regularly during pregnancy.	1	2	3	4	5
Other pregnant women are sufficiently informed about maintaining oral health.	1	2	3	4	5
During pregnancy, women seek more information about oral health.	1	2	3	4	5

During pregnancy, women pay more attention to their oral health.	1	2	3	4	5
During pregnancy, pregnant women take supplements that support the child's dental health.	1	2	3	4	5
Because teeth are more sensitive in pregnancy, most women attend dental checkups more often.	1	2	3	4	5
